# Novel Molecular Targets of Antidepressants

**DOI:** 10.3390/molecules27020533

**Published:** 2022-01-14

**Authors:** Małgorzata Jarończyk, Jarosław Walory

**Affiliations:** National Medicines Institute, 30/34 Chełmska Str., 00-725 Warsaw, Poland; jwalory@gmail.com

**Keywords:** antidepressants, GPCR, monoamine transporters, drug discovery

## Abstract

Antidepressants target a variety of proteins in the central nervous system (CNS), the most important belonging to the family of G-protein coupled receptors and the family of neurotransmitter transporters. The increasing number of crystallographic structures of these proteins have significantly contributed to the knowledge of their mechanism of action, as well as to the design of new drugs. Several computational approaches such as molecular docking, molecular dynamics, and virtual screening are useful for elucidating the mechanism of drug action and are important for drug design. This review is a survey of molecular targets for antidepressants in the CNS and computer based strategies to discover novel compounds with antidepressant activity.

## 1. Introduction

Major depressive disorder (MDD) is a serious and, in severe cases, a lethal disorder. Despite extensive research, the knowledge about the pathophysiology, specific mechanisms, and regulatory pathways underlying the disease remains limited [1]. Based on clinical effects and molecular mechanism of antidepressant drugs, the monoamine hypotheses were proposed more than 50 years ago, suggesting that depression pathophysiology is associated with deficiencies of the monoamine neurotransmitter serotonin (5-HT), dopamine (DA), and norepinephrine (NE) [2]. However, it is evident that monoamine-deficiency only partly explains the pathogenesis, and other neurotransmitters including acetylcholine, glutamate, and gamma-amino butyric acid (GABA) have also been connected to the etiology of depression. The monoamine deficiency may rather be a consequence than a cause of the disorder [3,4]. Although numerous antidepressant drugs are on the market, effective management of MDD is still problematic. Several drugs such as selective serotonin reuptake inhibitors (SSRI) and serotonin-norepinephrine reuptake inhibitors (SNRI), older tricyclic antidepressants (TCA), and a growing number of other types of drugs are used as antidepressants. However, delayed onset of action and undesirable side effects are common. Furthermore, approximately 50% of depressed patients achieve complete remission despite optimized treatment, including trials of multiple drugs with and without simultaneous psychotherapy. Therefore, there is an urgent need to develop new approaches to obtain more effective, safer, and faster antidepressants.

A better understanding of pathogenic processes involved in depression is needed to develop improved therapeutics. The monoamine-depletion hypothesis alone cannot fully explain the pathogenesis of depression. Depression is a complex disease associated with dysregulation of the immune system and the hypothalamic-pituitary-adrenal (HPA) axis, as well as neurotransmitter and neurotrophic systems imbalance [5,6]. The “inflammatory hypothesis” of depression was proposed more than two decades ago and suggests that inflammatory processes are involved in the onset and maintenance of MDD [7,8]. The increased concentrations of circulating pro-inflammatory cytokines such as interleukin-6 (IL-6), tumor necrosis factor-α (TNF-α), and interleukin-1β (IL-1β) were found in depressed patients. IL-1β is one of the most potent pro-inflammatory cytokines secreted by activated inflammatory cells that was found to play an important role in depressive-like behaviors as well as stress-related cellular actions. In the central nervous system (CNS), a key player involved in the secretion of biologically active IL-1β is P2X7R, an ATP-gated ion channel present on immune cells. Pharmacological targeting of P2X7R might have the potential as a future therapy for the treatment of a subset of patients with depression [9]. Hyperactivity of the HPA-axis has also been implicated in the pathophysiology of depression. There are two glucocorticoid receptors in the brain that control the HPA axis: the mineralocorticoid receptor (MR) and the glucocorticoid receptor (GR). Hence, targeting these receptors by antidepressants was found to normalize the HPA-axis and is considered a promising strategy for the development of novel antidepressants drugs [10]. Reduced levels of brain-derived neurotrophic factor (BDNF) lead to depression, according to the neurotrophic hypothesis of depression [11,12]. Other neurotrophic/growth factors linked to depression include the vascular endothelial growth factor (VEGF), the fibroblast growth factor 2 (FGF2), and the insulin-like growth factor 1 (IGF-1).

G-protein coupled receptors (GPCRs) and neurotransmitter transporters play well-established roles in the pathophysiology of depression and are targets for present antidepressant drugs, and are also recognized targets in the search for more specific and effective pharmacological approaches. In the present review, we will focus on GPCRs and neurotransmitter transporters in the CNS as targets for antidepressants.

## 2. GPCR as Targets for Antidepressants

GPCRs represent the largest class of membrane receptors in humans, containing seven membrane-spanning helices. They transduce extracellular signals into the cell interior through coupling to heterotrimeric G proteins and activation of different signaling pathways. Members of GPCRs from the different receptor families have been implicated in depression (see review [13]). The most important GPCR targets, antidepressant compounds and signaling pathways activated by target proteins, are listed in Table 1.

**Figure 1:**
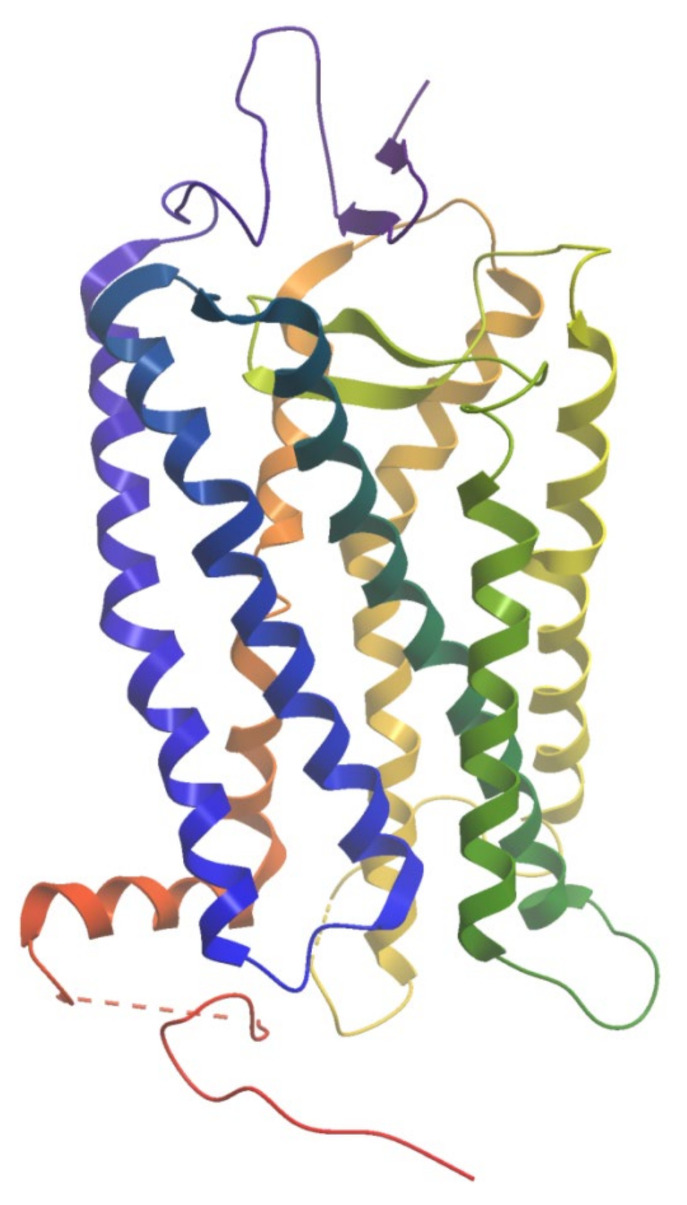
The structure of bovine rhodopsin (PDB ID: 1F88). The helices are colored from N-terminus (blue) to C-terminus (red).

The first GPCR structure at an atomic resolution was the structure of bovine rhodopsin in its inactive state (Figure 1), which was released in 2000 [14]. X-ray crystallography and cryo-electron microscopy (cryo-EM) technology enabled for GPCR structure determination and significantly deepened our knowledge of molecular mechanisms of signal transduction. At present, 455 structures representing 82 different receptors, are deposited in the Protein Data Bank (PDB) [15]. Structural studies of GPCRs have provided insight on the arrangements of transmembrane domains, the location of orthosteric, allosteric, bitopic, as well as biased ligand binding sites, and conformational changes upon GPCR activation and inactivation [16]. Structural knowledge of GPCRs have also given the possibility of structure-based drug design for obtaining new and improved compounds targeting the receptors. 

### 2.1. Serotonin Receptors

Serotonin receptors are found throughout central and peripheral nervous systems in the brain, mainly in regions involved in the neurobiology of anxiety and depression. There are 7 families of 5-HT receptors (5-HT1–5-HT7), which are further subdivided into 14 distinct receptor subtypes. Except for the 5-HT3 receptor, which is a ligand-gated ion channel, all known 5-HT receptors are G-protein coupled [17].

One of the most important and extensively studied 5-HT1 subtypes is the 5-HT1A receptor due to its implication in the pathophysiology of several neuropsychiatric disorders including anxiety and MDD [18]. The 5-HT1A receptors are distributed in the limbic, cortical, and dorsal and median raphe nucleus. The 5-HT1A receptors couple to the Gi/Go pathways, inhibiting the adenylyl cyclase to reduce cyclic adenosine monophosphate (cAMP) level and activating the G-protein inward rectifying potassium (GIRK) channels [19]. 5-HT1A function as presynaptic autoreceptors and postsynaptic heteroreceptors and signal to diverse and sometimes opposing pathways [19]. Identifying biased 5-HT1A ligands that preferentially activate one pathway over another may offer novel strategies for depression treatment [20]. Several 5-HT1A receptor agonists such as buspirone and tandospirone are medications approved to treat anxiety and depression [21]. Moreover, biased ligands of 5-HT1A such as F-15599 and F-13714 activate selectively postsynaptic heteroreceptors and presynaptic autoreceptors, respectively [22,23].

Until recently, the atomistic structure of the 5-HT1A receptor was not resolved. Thus, homology models of 5-HT1A receptor were built based on the bovine rhodopsin template [14], which after a while was replaced by the β2AR crystal structure [24]. Later, the β1-adrenergic [25] and the A2A adenosine [26] receptor crystal structures were also used as templates. However, during the last years the crystal structure of the 5-HT1B receptor has been the most used template for 5-HT1A receptor models [27]. Recently, the cryo-EM structures of the 5-HT1A in complex with the Gi subunit were determined at a resolution of 3.0–3.1 Å [28]. These structures reveal insight in the primary activity of the 5-HT1A receptor and the mode of drug recognition.

### 2.2. Dopamine Receptors

The dopamine system plays an important role in the pathogenesis of depression, and efficacy of dopamine receptor ligands in the treatment of human depression have been reported [29]. Dopamine receptors are grouped into two families: D1-like receptors (D1- and D5-receptors) and D2-like receptors (D2-, D3-, and D4-receptors). The D2-like receptors (D2/D3) play a key role in the response to antidepressant treatment [30]. This group of receptors have been found mainly in the striatum, amygdala, cerebral cortex, hippocampus, and pituitary gland [31]. They couple to Gαi/o proteins and primarily inhibit adenylate cyclase.

Aripiprazole is a D2 partial agonist that was initially approved for the treatment of schizophrenia, and later as an augmenting agent in MDD [32]. The brexpiprazole is a partial D2 agonist that has lower intrinsic activity than aripiprazole but higher 5-HT1A/2A receptor binding affinity [33]. The compounds with high affinity and occupancy of both D2 and D3 dopamine receptors may be effective in the treatment of depressive disorders and schizophrenia [34]. Cariprazine is a potent dopamine D2/D3 receptor partial agonist that was found to be effective in the treatment of depression [35]. Other receptor interactions are also implicated in the antidepressant effects of cariprazine, notably 5-HT1A receptor agonism.

The resolved crystal structures of the D3 and D2 receptors are helpful for understanding their mechanisms of action and their key interactions with the ligand in the binding site [36,37]. Studies to identify potent, novel, and selective dopamine D2 and D3 receptor ligands have been performed and novel potent compounds have been synthesized and tested using in vitro and in silico methods [38].

### 2.3. Opioid Receptors

A novel target for the treatment of depression may be the endogenous opioid system, which consists of mu (μ), kappa (κ), and delta (δ) opioid receptors (MORs, KORs, DORs) and the non-opioid receptor, nonciceptin (NOP), previously referred to as opioid-like 1 receptor. Several studies concerning the role of opioid receptors in depression treatment have emerged [39,40,41]. Opioid receptors are widely distributed in the hippocampus, nucleus accumbens, prefrontal cortex, amygdala, claustrum, thalamus, hypothalamus, ventral tegmental area and dorsal raphe nucleus [42]. Opioid receptors are coupled to inhibitory heterotrimeric Gαi/o proteins and also stimulate G protein-independent signaling pathways, notably via β-arrestins.

Buprenorphine is a partial agonist of μ opioid receptor and κ opioid receptors, and has activity at DOR and NOP receptors [43]. In preclinical studies, the antidepressant effects of buprenorphine were shown to be mediated via κ opioid receptors [44]. Furthermore, in vivo studies and behavioral tests indicated that nalmefene (NMF), a partial κ opioid receptor agonist and potent μ opioid receptor antagonist, has antidepressant activity [45]. Tianeptine is a MOR agonist and activate signaling pathways different to that of morphine [46]. A specific blockade of NOP receptors has been suggested to induce antidepressant-like action in preclinical tests [47]. The NOP receptor antagonist BTRX-246040 (known as LY-2940094) displays antidepressant-like effects in rodents models [48,49].

High-resolution crystal structures providing insight into molecular determinants required for ligand binding to opioid receptors were resolved: μ receptor [50,51], δ receptor [52], κ receptor [53], and NOP receptor [54,55]. Recently a 3.1-Å resolution X-ray structure of the KOR in the activated state bound to the high-affinity agonist MP1104 was reported [56]. Computational studies using the crystal structure of NOP receptor gave insight into the binding mode of two novel NOP antagonists, one selective (BTRX-246040), and one unselective (AT-076) antagonist [57].

### 2.4. Glutamate Receptors

Targeting the glutamatergic system may be a promising strategy for developing new treatments in mood disorders [58]. The glutamate receptors are divided into two groups: the ionotropic glutamate receptors and G-protein coupled metabotropic glutamate receptors (mGluRs). The first group includes N-methyl-D-aspartate (NMDA), α-amino-3-hydroxy-5-methyl-4-isoxazolepropionic acid (AMPA) and kainite receptors. The mGluRs are located presynaptically and postsynaptically, and are classified into three subgroups based on sequence similarity, G-protein coupling, and ligand selectivity. Receptors of subgroup I (mGlu1 and mGlu5) couple to Gq/G11 and activate phospholipase Cβ, while receptors of subgroup II (mGlu2 and mGlu3) and subgroup III (mGlu4, mGlu6–8) couple to Gi/o proteins, thus leading to adenylyl cyclase inhibition. Subgroup II of mGluRs is an attractive target for the development of novel antidepressants, as confirmed by preclinical studies [59]. The mGluR2 is expressed only in cerebellar cortex and olfactory bulbs while the mGluR3 is extensively detected in dentate gyrus, cerebral cortex, striatum, substantia nigra pars reticulata, olfactory tubercle, and lateral septal nucleus [60]. Preclinical studies indicated that two mGlu2/3 receptor antagonists (MGS0039 and LY341495) produced antidepressant effects [61,62]. Moreover, the negative allosteric modulator of mGlu2/3 receptors, RO4491533, was found to have antidepressant-like effects in mice in the forced swim test (FST) [63].

All mGlu receptors exist as homodimers possessing an extracellular Venus flytrap (VFT) domains, which is linked via cysteine rich-domains (CRDs) to their 7-transmembrane (TM) domain. The 7TM domain of mGlu receptors is the binding site of various non-endogenous allosteric ligands that can modulate signaling either on their own or in conjunction with orthosteric ligands, thereby acting as positive allosteric modulators (PAMs), or negative allosteric modulators (NAMs). Crystal structures of mGlu1 [64] and mGlu5 [65] 7TM domains show that the ligand binding site of allosteric modulators largely overlap with that of orthosteric ligands in family A GPCRs. Computational and experimental studies of mGlu2 allosteric modulators based on the crystal templates of the mGlu1 [64] and mGlu5 [65] were performed to study binding modes of known NAMs and PAMs [66].

### 2.5. Orphan Receptors

The ‘orphan’ GPCRs are examples of genes without known functions. The expressed proteins share the structural similarity of seven transmembrane helices with other GPCRs but are called ‘orphans’ because their endogenous ligands have not been identified yet. Recent findings show that orphan GPCRs (oGPRs) may be implicated in depression and be putative targets for new drug development [67,68]. No crystal structures of the oGPRs are available yet, although homology models are deposited in the GPCR database [69].

GPR26 is an orphan GPCR expressed in the hippocampus, amygdala, and thalamus of the human brain [70]. GPR26 is coupled to Gs and activates the adenylyl cyclase pathway. Behavioral tests on GPR26 knockout mice indicated that GPR26 is important in regulating anxiety- and depression-like behaviors [71].

GPR56 is involved in a number of biological functions such as myelin formation, neurogenesis, and oligodendrocyte development. In vivo experiments indicated reduction of GPR56 expression in the prefrontal cortex (PFC) and dorsal hippocampus, which can be reversed after antidepressant treatment [72]. It was found that protein kinase B (AKT), glycogen synthase kinase 3 (GSK3), and eukaryotic initiation factor 4E (EIF4) pathways involved in depression were upregulated in cells after GPR56 agonists treatment such as peptides P7 “TYFAVLM-NH2” and P19 “TYFAVLMQLSPALVPAELL-NH2” [72]. In summary, it is suggested that GPR56 may represent a potential molecular target for the treatment of depression. 

GPR158 is highly upregulated in PFC area and plays an important role in the regulation of depression [73]. GPR158 lacks the ligand binding VFT domain, but it has conserved amino acids involved in G protein binding. The plasma membrane GPR158 acts as an anchor for regulating G protein signaling 7 (RGS7) complexes, thus may modulate the signaling of other GPCRs.

### 2.6. Trace Amine-Associated Receptor

In the CNS, trace amines (TAs) such as tyramine, β-phenylethylamine (β-PEA), octopamine, and tryptamine were found to play an important role as neurotransmitters. Their dysregulations are implicated in the pathophysiology of neuropsychiatric disorders such as depression and schizophrenia. Clinical studies reported decreased urinary excretion of β-PEA in patients with depressive disorders and PEA administration produced long-lasting relief of depression in a patient population [74,75]. In 2001, the trace-amine-associated receptors (TAARs) were discovered and classified into the three subfamilies of TAARs 1–4, TAAR5, and TAARs 6–9 [76]. The most studied of these receptors is TAAR1, which could be activated by different biogenic amines and psychoactive compounds [77]. TAAR1 is widely expressed across the mammalian brain, particularly in cerebral cortex and hippocampus and signals through stimulatory Gs proteins to elevate intracellular cAMP levels and stimulate inwardly rectifying K+ channels [78,79]. In behavioral animal studies, the partial agonists of TAAR1, RO5203648 demonstrated potential antidepressant-like properties [80]. Furthermore, the studies of RO5263397, TAAR1 agonist demonstrated a response in the forced swim test, a rodent model of depression-like behaviors [81].

The second most studied member of the TAARs family, TAAR5, was initially identified as a putative neurotransmitter receptor (PNR) and expression of TAAR5 mRNA was reported in the human amygdala, the hippocampus, the caudate nucleus, the thalamus, the hypothalamus, and the substantia nigra and skeletal muscles [82]. Recent animal studies revealed involvement of this receptor in the pathogenesis of neuropsychiatric diseases [83]. The TAAR5 knockout mice had elevated levels of dopamine and its dopamine metabolites and altered levels of serotonin in the brain [84]. The human TAAR5 signals through Gs, the Gq/11, or G12/13-dependent mitogen-activated protein kinase pathways. Trimethylamine is one of the known agonists of TAAR5, but until now antagonists have not been discovered.

Crystal structures of trace amine receptors have not been resolved yet. However, homology models of TAAR1 were built based on the available crystal structures of human β2-adrenoreceptor to identify novel ligands of this receptor and key amino acids responsible for ligand binding [85,86]. The murine and human homology models of TAAR5 were built based on the crystal structures of human β2-adrenoreceptor and virtual screening was performed in order to find novel ligands [87]. These studies identified two compounds acting as murine TAAR5 antagonists, which were further validated by experimental studies.

### 2.7. Cannabinoid Receptors

The importance of the endogenous cannabinoid system (ECS) in depression, composed of cannabinoid receptor types 1 and 2 (CB1R and CB2R), has been confirmed in preclinical and clinical studies. Bambico et al. [88] reported that at low doses, the CB1R agonist WIN55212-2 exerts potent antidepressant-like properties in the rat FST. An increase of CB1R density in the pre-frontal cortex and concomitant mediated signaling suggest a role of the endocannabinoid system in the etiology of depression [89]. Animal studies based on a mouse model with CB2R deletion in dopamine neurons showed that CB2Rs in dopamine neurons play a role in modulating depression- and anxiety-like behaviors [90,91]. The CB1 receptors are located in the central nervous system, particularly in the hippocampus, prefrontal cortex, basal ganglia, cerebellum, amygdala, spinal cord, and mesolimbic nuclei [92]. The CB2 receptors are mainly distributed in the immune system (the spleen, tonsils, immune system cells), but can also be found within the CNS in microglia [93]. CB1 and CB2 receptors are both Gi/o-coupled GPCRs, and their activation leads to the inhibition of adenylyl cyclase and reduction in the production of cAMP [94]. Since both cannabinoid receptors play an important role in mood regulation (see review [95]), studies of searching for novel pharmacological agents is needed. As an example, antidepressant-like behavioral properties of CB1R agonists such as Δ9-tetrahydrocannabinol (THC) and rimonabant, terpineol were confirmed in olfactory bulbectomy studies [96]. Among agents acting through CB2R, β-caryophyllene (BCP), a naturally available sesquiterpene, is pharmacologically a selective agonist for CB2 receptors that produced an antidepressant–like effect shown in the animal model of depression [97].

Recently the crystal structures of CB1 [98,99] and CB2 [100] receptors were published. The role of cannabinoid receptor signaling pathway in the antidepressant-like effect of terpineol was confirmed by using pharmacological and molecular modeling approaches [101]. Terpineol shares structural similarity with cannabinoid receptor ligands and showed a coherent predicted binding mode mainly against CB1 and CB2 receptors during docking analyses [101].

### 2.8. Cholinergic Receptors

In 1980, Janowsky et al. [102] and Dilsaver et al. [103] postulated that the cholinergic system is involved in the pathophysiology of depression. The role of both muscarinic and nicotinic cholinergic receptors in the mood regulation, based on human and animal studies, has been reviewed by Dulawa and Janovsky [104]. Nicotinic receptors are ligand gated ion channels, whereas muscarinic receptors belong to the super family of GPCRs. Activation of the muscarinic M1, M3, and M5 receptors stimulates a cascade of interactions, including G protein Gq/11 and phospholipase C activation, resulting in formation of the second messenger inositol triphosphate (IP3) from phospholipids, which subsequently induces a release of calcium.

The muscarinic M2 and M4 receptors couple through Gi and Go proteins, giving inhibition of adenylate cyclase activity and reduced formation of cAMP. The muscarinic antagonist scopolamine has been reported to have antidepressant-like effects in patients suffering from unipolar depression [105]. At first, it was found that scopolamine exerts its antidepressant effect by blockade of the M1 receptor, increasing the mechanistic target of rapamycin complex 1 (mTORC1) signaling in pre-frontal cortex [106]. Furthermore, the involvement of the M2 receptor in mediating antidepressant-like effects of scopolamine by increasing the expression of BDNF and activating the mTORC1 signaling pathway was confirmed [107]. Other antagonists such as VU0255035 also demonstrate their antidepressant effect by interaction with M1 or M2 receptors. 

For better understanding of the molecular mechanism of the action of muscarinic receptors, knowledge of the crystal structure of these receptors is needed. So far, the crystal structures of the M2 and M3 muscarinic receptors and the M1 and M4 muscarinic receptors have been resolved [108,109,110].

### 2.9. GABA Receptors

The γ-Aminobutyric acid (GABA) is the principal neurotransmitter mediating neural inhibition in the brain. There are two types of GABAergic receptors: the ionotropic receptors GABA-A and GABA-C, and the metabotropic receptor GABA-B. Located both pre- and postsynaptically, GABA-B receptors influence cAMP production through coupling to Gi and Go proteins. GABA-B receptors are formed by dimerization of two homologous subunits GABAB(1) and GABAB(2), composed of three domains: a long extracellular N-terminal domain called the Venus fly-trap domain (VFT), which contains the orthosteric binding site for GABA; a heptahelical transmembrane domain (7TM); and a C-terminal intracellular tail. The role of GABA-B receptors in depression was first proposed by Lloyd [111] and confirmed in several studies. In preclinical studies, the knockout mice lacking the GABAB(1) subunit demonstrated decreased immobility in FST while no difference in mice behavior was observed in tail suspension test (TST) [112]. Baclofen, a known agonist of the GABA-B receptor was found to be effective in the treatment of posttraumatic stress disorder in the clinical studies [113]. The GABA-B antagonist CGP56433A exerted an antidepressant-like effect by decreasing the immobility in the FST [112]. Other GABA-B antagonists such as CGP36742 and CGP51176 were also effective in inducing antidepressant-like effects in FST [114]. The positive allosteric modulators GS39783 and CGP7930 were found to modulate activity of GABA-B by decreasing the affinity of antagonist radioligand in saturation experiments [115].

Recently, the GABA-B receptor structure and activity and interactions with compounds were reviewed by Evenseth et al. [116]. The published structures resolved by cryo-electron microscopy [117,118,119] provide insight into GABA-B receptor mechanism of action and information of allosteric and orthosteric ligand binding sites and may lead to design new potent antidepressants.

### 2.10. Neurokinin Receptors

Neuropeptides such as substance P (SP) and neurokinin A and neurokinin B belong to the family of neurotransmitters known as tachykinin. There are three subtypes of neurokinin receptors: NK1, NK2, and NK3. Neuroanatomical studies demonstrated that the predominant tachykinin receptor is NK1R in the human brain, whereas the expression of NK2 or NK3 receptors is either weak or absent. The NK1Rs are widely expressed throughout the CNS in areas including the amygdala, hypothalamus, hippocampus, and striatum [120]. These regions are involved in regulating the stress response and controlling affective behavior, such as depression and anxiety. Neurokinin-1 receptor signals through the Gαq-protein and induces activation of phospholipase C followed by production of IP3, leading to elevation of intracellular calcium as a second messenger. Further, cyclic AMP is stimulated by NK1R coupled to the Gαs-protein. In the clinical studies of patients with MDD, Kramer et al. [121,122] observed antidepressant effects of the NK1 receptor antagonists, MK-869 (aprepitant) and L-759274.

Recently, the crystal structure of hNK1R bound to the antagonist L760735, a close analog of aprepitant, was solved [123]. In addition, three high-resolution crystal structures of the human NK1 receptor bound to two small-molecule antagonist therapeutics—aprepitant, netupitant, and the progenitor antagonist CP-99,994 were determined [124]. Conformational changes at the helix II-VII interface upon antagonist binding were observed. The understanding of molecular structures provides opportunity to develop ligands that will selectively target different neurokinin receptors.

### 2.11. Cholecystokinin Receptors

Cholecystokinin (CCK) is a gut–brain peptide that has been implicated in stress and anxiety disorders [125]. There are two types of receptors: CCK1 (CCK-A) receptors are present mainly in peripheral tissues and in discrete brain regions, while CCK2 (CCK-B) receptors predominate in the central nervous system. The CCKR2 is widely distributed in the brain, especially in the cerebral cortex, nucleus accumbens, caudate nucleus, hippocampus, amygdala, substantia nigra, and ventral tegmental area [126,127]. The neuronal co-localization of CCK receptors and the dopaminergic system reveal involvement of both systems in neuropsychiatric and CNS disorders [128]. Signal transduction at CCK1 receptors is mediated via Gs protein while at CCK1R via Gq protein and to a lesser extent with Gi.

A reduction in the suppression of motility in shocked mice was observed after treatment with L-365,260, a CCK2R antagonist, which strongly suggests that CCK2R antagonists are able to induce antidepressant-like effects [129]. The blockade of CCK2R by the selective antagonist CI-988 prevented an increase in serum corticosterone levels in animal tests and increased immobility time in the FST [130]. 

Recently, the cryo-EM structures of CCK1R in complex with sulfated cholecystokinin-8 (CCK-8) coupled to different G-proteins were determined [131]. Furthermore, the crystal structures of CCK1R in complex with antagonists (devazepide and lintitript) and CCK-8, and two cryo-EM structures of CCK2R with gastrin coupled to Gi and Gq proteins were resolved [132]. They are the basis for studying the mechanism of ligand binding using molecular modeling methods to develop new antidepressants.

### 2.12. G-Proteins and G-Protein Regulating Proteins

G-proteins play an important role in transducing signals from receptors into cell and may be involved in the pathogenesis and treatment of mood disorders (see review [133]). Firstly, the alterations in the concentration or function of G proteins in peripheral blood elements and in postmortem tissues of patients with bipolar and other mood disorders were observed after treatment with lithium [134,135]. Further studies with other antidepressant do not confirm changes in G protein expression [136,137].

Regulators of G protein signaling (RGS) proteins control GPCR and linked G protein signaling. They are implicated in the CNS disorders (see reviews [138,139]). Among RGS proteins, RGS2 was found to be expressed in brain areas important in the pathogenesis of anxiety and depression such as the hippocampus, amygdala, cerebral cortex, hypothalamus, and raphe nucleus [140]. The crystal structure of RGS2 in complex with Gαq was resolved [141]. The studies on RGS7 show that the loss of striatal RGS7 induces an anxiolytic-like and antidepressant-like phenotype [142,143]. The crystal structure of RGS7-Gβ5 dimer has been resolved in 2018 [144]. Other regulatory proteins such as RGS4, RGS6, and RGS8 are widely expressed in the brain regions involved in depression [145,146]. Further studies are needed to understand the potential role of RGS proteins in major depressive disorders and their mechanism of action.

## 3. Neurotransmitter Transporters as Targets for Antidepressants

Several transporters in the solute carrier 6 (SLC6) family mediate the uptake of released neurotransmitters from the extracellular space into neurons and glial cells. They are widely expressed in the mammalian brain and play an important role in regulating neurotransmitter signaling [147,148]. The three types of cell membrane monoamine transporters (MATs) are the dopamine transporter (DAT), norepinephrine transporter (NET), and the serotonin transporter (SERT). These transporters mediate the uptake of monoamine neurotransmitters dopamine, norepinephrine, and serotonin, respectively, from the extracellular space into the intracellular compartment [149]. They are the main targets for the present antidepressants and several drugs of abuse [150].

**Figure 2:**
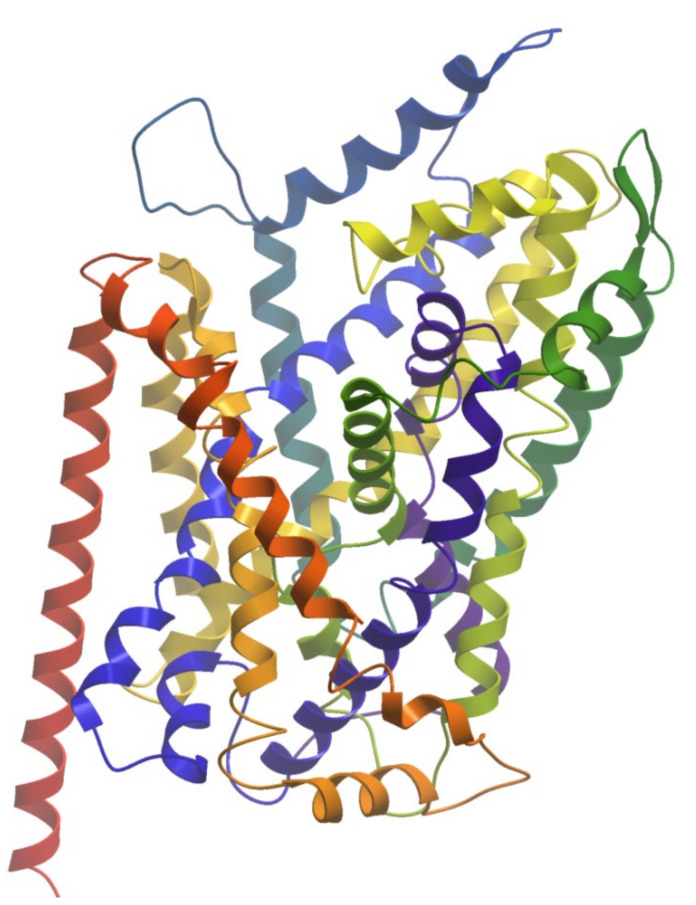
The structure of leucine transporter (PDB ID: 2A65). The helices are colored from N-terminus (blue) to C-terminus (red).

Human MATs are large integral membrane proteins consisting of about 600 amino acids that contain 12 transmembrane α-helices (TM1 to TM12) connected by intra- and extracellular loops (ILs and ELs). Crystal structures of transporter bound with substrate and inhibitors have advanced our understanding of the mechanism of action of MATs and help in the discovery of novel antidepressants. The first structure elucidated was a bacterial homologue of the human monoamine transporters, the leucine transporter (LeuT) bound to its substrate in an occluded conformation (Figure 2) [151]. Furthermore, several crystal structures of LeuT in different conformational states were reported [152,153,154,155]. In addition, crystal structures of the Drosophila melanogaster dopamine transporter (dDAT) [156] and human serotonin transporter [157] in complex with different transport blockers and in altered conformational states were resolved and have given remarkable insights into the inhibitory mechanisms of these transporters.

Selective serotonin reuptake inhibitors, serotonin-norepinephrine reuptake inhibitors, and serotonin-norepinephrine-dopamine reuptake inhibitors (SNDRI) are widely used antidepressants [158]. Furthermore, antidepressants that inhibit both norepinephrine and serotonin transporters have been developed. It has been suggested that these SNRIs have improved antidepressant efficacy and faster onset of action in comparison to SSRIs. Among these SNRIs are duloxetine and venlafaxine that increase 5-HT and NE levels specifically in the prefrontal cortex area of the brain as well as the dopamine levels. Selective norepinephrine reuptake inhibitors (NRIs) such as reboxetine and nisoxetine are also in use for depression treatment and attention deficit hyperactivity disorder (ADHD). Another group of antidepressant are dual NET/DAT inhibitors (e.g., nomifensin), while triple reuptake inhibitors (SERT/DAT/NET inhibitors, e.g., indatraline and mazindol) are being examined for their efficacy in depression and other CNS-disorders [158].

### 3.1. Serotonin Transporter

The SERT protein plays an important role in the serotoninergic system. The highest levels of this transporter are found in the raphe nuclei and the cerebellum, basal ganglia, and thalamus, followed by the hippocampus and the prefrontal cortex [159]. The first-generation of MAT inhibitors include the tricyclic antidepressants such as imipramine, clomipramine, desipramine, and amitriptyline [160]. They exhibit their antidepressant effects by increasing synaptic levels of 5-HT and NE via inhibition of SERT and NET. SSRIs are considered as the second generation of antidepressants, and block the reuptake of serotonin from the synaptic cleft. The most commonly prescribed SSRIs are paroxetine, fluoxetine, citalopram, and sertraline [161,162]. Despite their good safety profile and efficacy, the major disadvantage of SSRIs are their slow onset of action and side effects such as anxiety, sleep disturbances, and sexual dysfunction.

The recently reported X-ray structures of SERT in complex with (S)-citalopram, paroxetine, sertraline, or fluvoxamine now provides knowledge to help understand the structure activity relationship and selectivity of compounds relative to other monoamine transporters [157,163]. The cryo-EM structures of hSERT in complex with ibogaine in various conformational states were recently resolved [164]. The conformational dynamics of SERT transport function and inhibition is now extensively studied by using computational studies and hydrogen-deuterium exchange (HDX) experiments [165,166].

### 3.2. Dopamine Transporter

DAT belongs to MAT and controls neurotransmitter dopamine homeostasis in the brain by reuptake of DA. DAT is widely distributed through the brain in areas of dopaminergic activity, including the striatum and substantial nigra [167]. Numerous abused as well as clinically important drugs have important pharmacological interactions with DAT. Bupropion is a dopamine reuptake inhibitor and also an inhibitor of the norepinephrine transporter [168]. 

However, the molecular mechanism of action of the dopamine transporter has been elusive due to the lack of X-ray structure. The first reported was the crystal structure of the Drosophila melanogaster dopamine transporter (dDAT) in complex with TCA nortriptyline, then the X-ray structure of dDAT in complex with nisoxetine and reboxetine was resolved [156,169]. Furthermore, another dDAT crystal structure in complex with its substrate DA, as well as psychostimulants cocaine and amphetamine, was published [170]. Computational studies based on the crystal structures gave insight into the mechanism of interaction of drugs with DAT [171]. In addition, the potential significance of orphenadrine (ORPH), an inhibitor of NET reuptake, as a repurposable hDAT-inhibitor, was highlighted [171]. Recently, computational methods including molecular docking simulation and pharmacokinetics study were used for the identification of novel DAT inhibitors [124].

### 3.3. Norepinephrine Transporter

NET is a MAT which mediates the uptake of norepinephrine and is a drug target in major depression [172]. Radioligand studies indicated that binding of [3H] nisoxetine in the locus coeruleus (LC) is characteristic of binding to NET [173]. In vivo studies using positron emission tomography (PET) have reported higher norepinephrine transporter availability in the thalamus and its sub-regions in patients with major depressive disorder [172]. Reboxetine and nisoxetine are potent and selective NET inhibitors which have been successfully developed to treat depression and attention deficit hyperactivity disorder (ADHD).

For the development of more active and/or selective compounds, the knowledge of the crystal structure of the NET is needed for understanding the interactions with its ligands. However, the crystal structure of hNET is not available yet. Recent studies on molecular mechanism of action using homology models of hNET indicated amino acid residues responsible for inhibitor binding and may help to understand the molecular interactions between NET and its inhibitors [174,175].

## 4. In Silico Methods in Drug Design 

The advances in X-ray crystallography and cryo-EM methods have given new structural knowledge about the structure and function of GPCRs and MATs in the CNS and have given the opportunity of using target based (structure based) approaches for designing new compounds with a putative therapeutic value.

The basis for drug design is studying the action mechanism of a protein by determination of the correct binding conformation of small molecule ligands in the protein. Molecular docking is one of the most commonly used methods for predicting the conformation of small-molecule ligands within the appropriate target binding site with a high degree of accuracy [176]. Docking can provide theoretical calculations for target-ligand binding conformation and binding affinity scores, making it useful for both initial hit compound screening and computational analysis of lead compound binding patterns.

Understanding the structural dynamics and mechanisms of various GPCR signaling pathways is critical for the design of GPCR targeted ligands. GPCRs play critical roles in cellular signal transduction. The structural dynamics of GPCR signaling are supported by strong evidence that these receptors exist in multiple conformational states, contrary to their initial understanding as simple ON/OFF molecular switches [177]. Different agonists or allosteric modulators can stabilize different conformational states of the receptor, resulting in a signaling bias towards a specific G protein subtype or β-arrestin-mediated signaling pathways. Molecular dynamic simulations of GPCRs may demonstrate several “intermediate” conformational states that differ from the crystallographically observed active and inactive states. During substrate translocation, the monoamine transporters undergo conformational changes as well [178]. The established binding modes and behavior of neurotransmitters within MATs can greatly aid in structure-based development of novel drugs as well as in the ongoing optimization of existing drugs [179].

Virtual screening of large and chemically diverse compound libraries using computational methods is one of the most commonly used strategies in drug discovery [180]. Virtual screening approaches are divided into ligand-based and structure-based. The structure-based virtual screening (SBVS) method makes use of the target protein crystal structure, whereas the ligand-based virtual screening (LBVS) method makes use of the structural information and physicochemical properties of the chemical scaffold of known active and inactive molecules. Virtual screening techniques were employed to design small molecule modulators of monoamine transporters to orthosteric and allosteric sites [181]. The virtual screening approach can be used for drug repurposing (or repositioning) [182]. The drug repurposing is the methodology of developing new pharmacological indications for existing drugs and has been found as a cost-effective strategy. Among the now 13 reported drugs that have been repurposed for the treatment of depression or bipolar depression are ketamine, dextromethorphan, and scopolamine [183,184].

The main reasons for the high rates of drug failure in the later drug development stage are undesirable pharmacokinetics and toxicity of drug candidates. Pharmacokinetic properties such as absorption, distribution, metabolism, and elimination/excretion properties (ADME) as well as toxicity are important in the drug discovery process. Therefore, there is a need for predictive tools that can eliminate inappropriate compounds to save time and money. For ADMET parameters such as bioavailability, aqueous solubility, intestinal permeability, blood-brain barrier penetration, metabolism, and toxicity, several computational approaches are useful [185].

## 5. Conclusions

GPCRs and MATs are the most important present therapeutic targets for developing novel drugs for the CNS disorders. GPCRs adopt a large number of conformational states upon ligand binding that activate various signaling pathways. The most important is linking the activation of various signaling pathways with the pharmacological activity of the drug, thus investigating the role of individual signaling pathways in the pathogenesis of depression. MATs also undergo local conformational changes in response to ligand binding and releasing, such as the closing of a gate on one side while opening a gate on the other side, or by forming a transient occluded state by closing both ends. However, the mechanisms of depression are still not well understood, and thus studies on the mechanism of protein actions and signaling pathways activated in the CNS are needed and justified to find new selective antidepressants.

## Figures and Tables

**Table 1:** GPCR targets, compounds with antidepressant activity, and signaling pathways activated by the target proteins.

Receptor Family	Target	Compounds	Signaling Pathways
Serotonin receptors	5-HT1A	Buspirone, Tandospirone, F-15599, F-13714	Gi/Go pathways (cAMP)
Dopamine receptors	D2, D3	Aripiprazole, Brexpiprazole, Cariprazine	Gi/Go pathways (cAMP)
Opioid receptors	MOR, DOR, KOR	Buprenorphine, Nalmefene, Tianeptine, BTRX-246040	Gi/Go pathways (cAMP)
Glutamate receptors	mGlu2, mGlu3	MGS0039, LY341495, RO4491533	Gi/Go pathways (cAMP)
Orphan receptors	GPR26, GPR56, GPR158,	P7, P19	Gs pathways (cAMP) AKT, GSK3, EIF4 upregulation RGS7 regulation
Trace amine associated receptors	TAAR1 TAAR5	RO5203648, RO5263397, Trimethylamine	Gs pathways (cAMP)
Cannabinoid receptors	CB1, CB2	THC, Rimonabant, Terpineol, β-caryophyllene	Gi/Go pathways (cAMP)
Cholinergic receptors	M1, M2	Scopolamine, VU0255035	Gi/Go pathways (cAMP) Gq pathways
GABA receptors	GABA B	CGP56433A, CGP36742, CGP51176, GS39783, CGP7930	Gi/Go pathways (cAMP)
Neurokinin receptors	NK1	Aprepitant, L-759274	Gs pathways (cAMP)
Cholecystokinin receptors	CCK2	L-365,260, CI-988	Gq pathways
